# Abnormalities of cortical-limbic-cerebellar white matter networks may contribute to treatment-resistant depression: a diffusion tensor imaging study

**DOI:** 10.1186/1471-244X-13-72

**Published:** 2013-03-02

**Authors:** Hong-jun Peng, Hui-rong Zheng, Yu-ping Ning, Yan Zhang, Bao-ci Shan, Li Zhang, Hai-chen Yang, Jun Liu, Ze-xuan Li, Jian-song Zhou, Zhi-jun Zhang, Ling-jiang Li

**Affiliations:** 1 Mental Health Institute, The 2nd Xiangya Hospital, Central South University, No. 139 Renmin Zhong Road, Changsha, 410011, China; 2 Guangzhou Psychiatric Hospital, Affiliated Hospital of Guangzhou Medical College, Guangzhou, China; 3 Guangdong Mental Health Institute, Guangdong General Hospital, Guangzhou, China; 4 Key Laboratory of Nuclear Analysis, Institute of High Energy Physics, Chinese Academy of Sciences, Beijing, People’s Republic of China; 5 Department of Radiology, The Second Xiangya Hospital of Central South University, Changsha, Hunan, China; 6 The Department of Neuropsychiatry and Institute of Neuropsychiatric Research, Affiliated ZhongDa Hospital of Southeast University, Nanjing, China; 7 Chinese University of Hong Kong, Hong Kong, China

**Keywords:** Treatment-resistant depression, Diffusion tensor imaging, Fractional anisotropy, Voxel-based analysis method

## Abstract

**Background:**

White matter abnormalities can cause network dysfunction that underlies major depressive disorder (MDD). Diffusion tensor imaging (DTI) is used to examine the neural connectivity and integrity of the white matter. Previous studies have implicated frontolimbic neural networks in the pathophysiology of MDD. Approximately 30% of MDD patients demonstrate treatment-resistant depression (TRD). However, the neurobiology of TRD remains unclear.

**Methods:**

We used a voxel-based analysis method to analyze DTI data in young patients with TRD (n = 30; 19 males, 11 females) compared with right-handed, age- and sex-matched healthy volunteers (n = 25; 14 males, 11 females).

**Results:**

We found a significant decrease in fractional anisotropy (FA) (corrected, cluster size >50) in the left middle frontal gyrus (peak coordinates [−18 46–14]), left limbic lobe uncus (peak coordinates [−18 2–22]), and right cerebellum posterior lobe (peak coordinates [26–34 -40]). There was no increase in FA in any brain region in patients. We also found a significant negative correlation between mean regional FA values in the three areas and Beck Depression Inventory symptom scores.

**Conclusions:**

We found significant differences in white matter FA in the frontal lobe, limbic lobe and cerebellum between TRD patients and controls. These data suggest that abnormalities of cortical-limbic-cerebellar white matter networks may contribute to TRD in young patients.

## Background

Major depression is a common condition and a leading cause of disability worldwide [1]. Approximately 5% of American adults are affected by depression each year, 30% of whom fail to respond to two or more types of antidepressant, a phenomenon termed treatment-resistant depression (TRD) [2-7]. The pathogenesis of major depressive disorder (MDD) and the pathogenic mechanism of TRD remain unclear. Techniques such as magnetic resonance imaging (MRI), especially diffusion tensor imaging (DTI), have revealed white matter abnormalities in multiple psychiatric disorders [8-10]. The white matter forms the basis of anatomical connectivity, and disruption of this connectivity can result in brain dysfunction underlying various psychiatric disorders [11,12]. DTI is a useful tool for examining and quantifying white matter microstructure, including the orientation and integrity of white matter tracts, by detecting the diffusion of water in neural tissue *in vivo*[10]. A high fractional anisotropy (FA) reflects intact axonal membranes, myelin sheaths, and a parallel arrangement of neurofibrils. By contrast, a low FA reflects damaged integrity of the white matter [9].

Previous studies using DTI have mainly focused on affective disorders including MDD, BD [13,14], and young and geriatric depression [15,16], and the results showed the abnormal brain regions include the superior, middle, and medial frontal gyrus [9,16,17], the subgenual anterior cingulate cortex (ACC), amygdala [14], hippocampus [18], and basal ganglia [19]. These abnormal brain regions are predominantly located in the limbic-cortical-striatal-pallidal-thalamic tract (LCSPT) [20,21], which is considered related to emotional behavior on the basis of its anatomical connectivity with visceral control structures that mediate emotional expression [19].

It remains unclear whether the pathogenesis of TRD is similar to various affective disorders, although there is some limited DTI evidence that abnormal brain areas in TRD include the LCSPT circuits, similar to general affective disorders [22]. In the present study, we used an explorative voxel-based analysis (VBA) method to investigate the white matter integrity of TRD patients in order to determine the specific microstructure alterations in TRD. We hypothesized that the changes in white matter FA in TRD are similar to general affective disorders involving abnormalities of the cortical-limbic or cortical-subcortical circuits, as well as other important areas related to emotional regulation.

## Methods

### Subjects

Thirty patients (mean age, 26.87 ± 5.28 years; mean disease course, 4.68 ± 3.37 years) fulfilled both our diagnostic criteria for a major depressive episode (DSM-IV) and the TRD criteria. We defined treatment resistance as failure to respond to at least two different classes of antidepressant given for a period longer than 4 weeks at the maximum recommended dose [23]. The patients were recruited from the inpatient and outpatient units at the Institute of Mental Health at the Second Xiangya Hospital of Central South University, and the sex- and age- matched healthy controls were recruited in the local community. After each subject was fully informed of the study, written informed consent was obtained. The protocol was approved by the Central South University ethics committee and the studies were carried out in accordance with the Declaration of Helsinki. Two experienced psychiatrists performed patient diagnosis independently. We excluded patients with other psychiatric axis-I or axis-II disorders, neurological disorders, and other clinically relevant abnormalities in laboratory examinations. The patients with a counter indication of MRI were also excluded. The Beck Depression Inventory (BDI) [24] was used to assess clinical symptoms.

### Diffusion tensor imaging data acquisition

The DTI scans were performed at the Magnetic Centre of Hunan Provincial People’s Hospital. Subjects wore a standard birdcage head coil when they lay supine in a 3.0-Tesla head scanner (Allegra, Siemens Medical System). We used foam pads to minimize head motion, and used ear-plugs to diminish the sounds of the scanner. We collected high-resolution T1-weighted whole-brain 3-D MRI data with a magnetization-prepared rapid-acquisition gradient echo sequence (MP-RAGE) using the following parameters: 144 sagittal slices; thickness, 1.0 mm; 256 × 256 matrix; field of view, 256 × 256 mm; TE, 3.7 ms; and TR, 2000 ms. We also collected a diffusion-weighted data set with an echo planar image sequence using the following parameters: 45 transversal slices; 30 gradient directions; thickness, 3.0 mm; no gap; 192 × 192 matrix; field of view, 240 × 240 mm; TE, 93 ms; TR, 6046 ms; b1, 0; and b2, 1000 s/mm^2^.

### Magnetic resonance imaging data analysis

Diffusion tensor images were pre-processed using previously published methods[25-27]. The diffusion data set was pre-aligned to correct for head motion, and the effects of gradient coil eddy currents were corrected using software tools from the FMRIB software library (http://www.fmrib.ox.ac.uk/fsl). The resulting FA images were transformed into Montreal Neurological Institute standard space using Statistical Parametric Mapping (SPM2; Wellcome Department of Cognitive Neurology, London, UK). For each subject, the b = 0 images were coregistered with the structural T1 image; the same coregistration parameters were applied to the FA maps (in the same space as the b = 0 images). Each individuals’ T1 image was then normalized to the SPM T1 template (in Montreal Neurological Institute standard space), and the same normalization parameters were then applied to the coregistered FA images. All images were resampled with a voxel size of 2 × 2 × 2 mm^3^. The normalized FA images were smoothed with an 8 mm full-width at half-maximum Gaussian kernel to decrease spatial noise, and a mean image (FA template) was created.

### Statistical analysis

Two-sample t-tests were performed between 30 TRD patients and 25 healthy controls on diffusion tensor images of FA using SPM2 software. An initial threshold of 50 voxels or greater, surviving a false discovery rate (FDR) threshold of *P* < 0.05, was set [28]. We retrieved white matter FA values from these identified clusters with home-developed software, as previously published [29]. Data were analyzed using SPSS17.0 software. A multiple-correlation analysis was performed to estimate the relationship between the average FA values and BDI scores, age, and duration of disease. A statistical threshold of *P* < 0.05 (two-tailed) was used.

## Results

### Clinical and demographic characteristics of the subjects

There were no significant age, gender, or marriage-state differences between patients and healthy control subjects (*P* > 0.05) (see Table 1).

**Table 1:** Clinical and demographic characteristics of patients TRD and HC

**Variable**	**HC (n = 25)**		**TRD group (n = 30)**		**P-value**
	**Mean**	**SD**	**Mean**	**SD**	
**Age(y)**	28.24	4.98	26.77	5.28	0.29
**Gender (male/female)(n)**	14/11		19/11		0.80
**Marriage (single/married)(n)**	15/10		16/14		0.92
**Course(y)**			4.68	3.37	
**BDI**			20.47	4.45	

P > 0.05. *HC*, healthy control; *SD*, standard deviation; *TRD*, treatment-resistant depression.

### Diffusion tensor imaging of treatment-resistant depression patients

**Figure 1:**
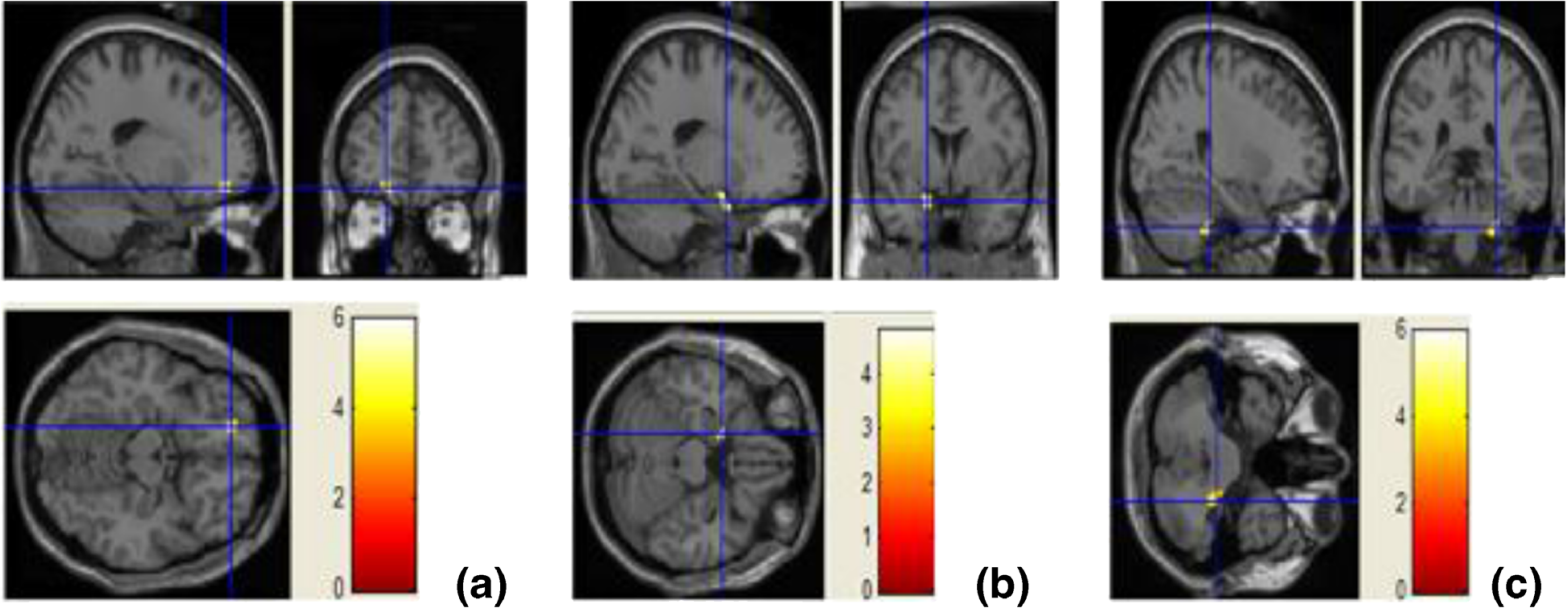
Areas of decreased fractional anisotropy extending over the left middle frontal gyrus (a), left limbic lobe uncus (b), and right cerebellum posterior lobe (c) in treatment-resistant depression patients compared with healthy control subjects.

**Figure 2:**
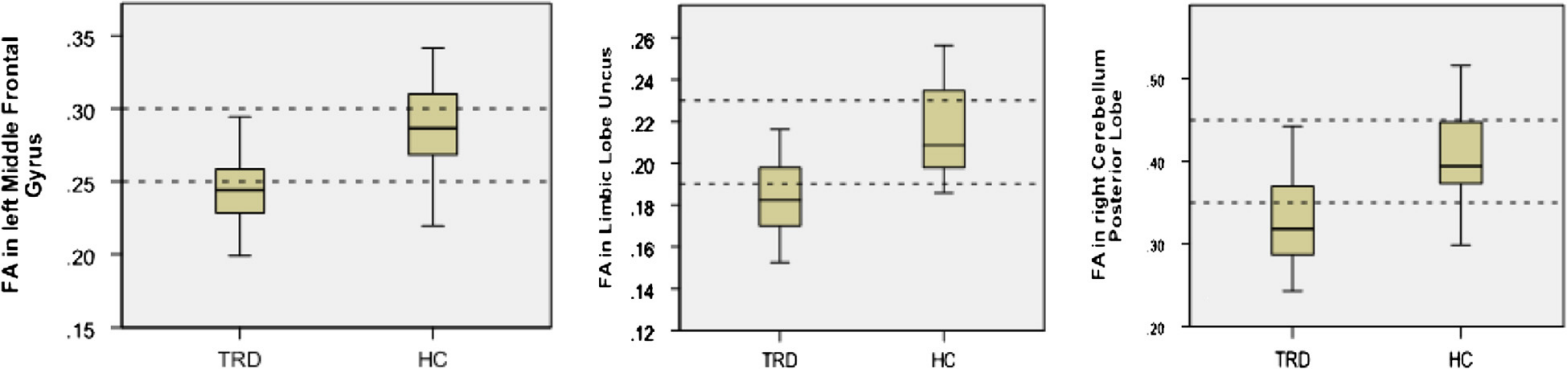
**Fractional anisotropy values are significantly decreased at the left middle frontal gyrus, left limbic lobe, and right cerebellum posterior lobe.***P* < 0.001. HC, healthy control; TRD, treatment-resistant depression.

Voxelwise analysis revealed reduced FA in three areas in the TRD group compared with control subjects (*P* < 0.001, uncorrected, cluster size > 50). One area was located at the left limbic lobe uncus with peak coordinates [−18 2–22], the second area was located at the left middle frontal gyrus [−18 46–14], and the third area was located at the right cerebellum posterior lobe [26–34 -40]. The three areas survived an FDR threshold of *P* < 0.05 at the cluster level or the voxel level (Figure 1 and Table 2). The left middle frontal gyrus survived FDR correction at the voxel level (*P* = 0.018), and the other two areas survived correction at the cluster level (*P* = 0.015 and *P* = 0.025, respectively). There were no other regions of reduced or increased FA of statistical significance in the TRD group compared with the control group. Our results showed significantly reduced FA values in the left middle frontal gyrus, left limbic lobe uncus, and right cerebellum posterior lobe in TRD subjects compared with controls (*P* < 0.001; see Figure 2).

**Table 2:** Brain regions with significantly lower fractional anisotropy

**Anatomical region**	**L/R**	**Cluster level P(corrected)**	**Size (voxels)**	**Voxel level P (FDR-corrected)**	**Voxel level P (uncorrected)**	**MNI (mm) x y z**			**Voxel Z**
Limbic lobe uncus	Left	0.015	92	0.232	0.000	−18	2	−22	4.13
Middle Frontal gyrus	Left	0.083	66	0.018	0.000	−18	46	−14	5.21
Cerebellum posterior Lobe	Right	0.025	89	0.125	0.000	26	−34	−40	3.98

Uncorrected *P* < 0.001, 50 voxels minimum extent. FDR, false discovery rate; MNI, Montreal Neurological Institute.

### Correlation between depressive symptom scores and fractional anisotropy values in treatment-resistant depression patients

**Figure 3:**
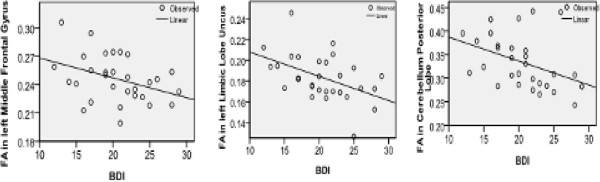
**Significant negative correlation between BDI scores of TRD patients and reduced FA values in the left middle frontal gyrus, left limbic lobe, and right cerebellum posterior lobe.** BDI, Beck Depression Inventory; FA, fractional anisotropy; TRD, treatment-resistant depression.

Significant negative correlations were found between depression symptom scores (BDI) and reduced FA values in the left middle frontal gyrus, right limbic lobe uncus, and right cerebellum posterior lobe regions of interest (Figure 3); the correlation coefficients were −0.379 (*P* = 0.039), -0.46 (*P* = 0.009), and −0.450 (*P* = 0.027), respectively. In addition, Pearson correlations found no correlations between FA values in regions of interest of TRD subjects, and age and disease duration.

### Effects of gender on reduced white matter fractional anisotropy values in the three regions

There were no significant differences between males and females for reduced FA values in the left middle frontal gyrus (*P* = 0.588), left limbic lobe uncus (*P* = 0.636), and right cerebellum posterior lobe (*P* = 0.207; see Table 3).

**Table 3:** Gender differences of mean fractional anisotropy in regions of interest

**Anatomical region**	**Gender**	**n**	**Mean**	**SD**	**P-value**
Left limbic lobe uncus	male	19	0.182	0.022	
	female	11	0.186	0.023	0.636
Left middle frontal gyrus	male	19	0.248	0.021	
	female	11	0.243	0.030	0.588
Right cerebellum posterior lobe	male	19	0.324	0.055	
	female	11	0.351	0.055	0.207

*P* > 0.05. SD, standard deviation.

## Discussion

We found significant differences in white matter FA between TRD patients and healthy subjects in the left middle frontal gyrus, left limbic lobe uncus, and right cerebellum posterior lobe. These data suggest that abnormal cortical-limbic-cerebellar white matter circuits may underlie the pathogenesis of TRD, which is partly consistent with previous studies on affective disorders that implicate abnormalities of the cortical-limbic circuits.

White matter abnormalities in the middle frontal gyrus and limbic lobe have been reported in numerous studies using the VBA or TBSS methods, including MDD, BD, young and geriatric depression, and first-episode and recurrent depression [12,14-17,30-35]; in these studies, abnormal cortical-limbic or cortical-subcortical circuits related to emotional regulation were used to interpret the mechanisms of affective disorders. In the cortical-limbic model proposed by Mayberg [36], the dorsal compartment includes both neocortical and midline limbic elements, and is thought to regulate attentional and cognitive symptoms of depression involving apathy and psychomotor retardation, while the ventral compartment, composed of the limbic, paralimbic cortical, subcortical, and brainstem regions, is proposed to mediate the vegetative and somatic aspects of depression. Depression is considered to be related to failure of the coordinated interactions of the dorsal and ventral compartment [37-39]. In our study, the left middle frontal gyrus and left limbic lobe uncus belong to the dorsal and ventral compartments, respectively, and dysfunction of these two compartments can account for the disturbances of emotional behavior. Modern brain imaging studies have supported a pronounced role of cortical-limbic top-down mechanisms in the regulation of mood and depression therapy, including the positive effect of cognitive behavioral therapy on depression [40,41].

The prefrontal cortex exerts a potent regulatory influence over the subcortical systems involved in the regulation of affective states [42,43]. Frontal-subcortical circuits such as the classic limbic-cortical-striatal-pallidal-thalamic (LCSPT) circuit, formed by connections between the orbital and medial prefrontal cortex, amygdala, hippocampal subiculum, ventromedial striatum, mediodorsal, and midline thalamic nuclei, and ventral pallidum, are considered to underlie emotional regulation [20,21]. These circuits can provide forebrain modulation over visceral control structures in the hypothalamus and brainstem, and their dysfunction can regulate the disturbances in autonomic regulation and neuroendocrine responses that are associated with mood disorders [8,20,44]. Our results showed abnormal white matter areas in the middle frontal gyrus and limbic lobe uncus, and the abnormal brain regions were located at or near LCSPT circuits. However, we did not find abnormalities in the classical brain areas such as the ACC, amygdala, or hippocampus often reported by previous studies of affective disorders [14,45], which may be related to our small sample size or rigorous threshold setting.

Besides the cortical-limbic or cortical-subcortical circuits, the cerebellum may also play an important role in emotional regulation. The traditionally held view is that the core functions of the cerebellum involve coordination, balance, and the motor component of speech regulation [46,47]. Recently, neuroanatomical studies have shown that the cerebellum is important for cognitive regulation through bidirectional pathways between the cerebellum and cortical structures, and cerebellar lesions can result in cerebellar-cognitive-affective syndrome, including executive, visual spatial, and linguistic impairments, and affective dysregulation. The cerebellum has extensive anatomic connections with many brainstem and forebrain structures. Several cerebellar-cerebral pathways are likely to be involved in emotional behavior, with several pathways emanating primarily from the cerebellar fastigial nuclei and terminating in various limbic structures including the hippocampus, amygdala, septal nuclei, mammillary bodies, and hypothalamus. Other potentially important pathways emanate from the ventrolateral dentate nucleus, travel to the thalamus (including dorsomedial nucleus), and terminate in the prefrontal cortex [48]. Doron *et al.* (2009) tracked connections between the cerebral peduncle and left hemispheric masks of the superior frontal gyrus, precentral gyrus, middle frontal gyrus, orbital frontal cortex, and two regions of the inferior frontal gyrus, supporting the relationship of the cerebellum with cognition and affection regulation [49]. In addition, the vermis of the cerebellum is recognized as an anatomical part of the limbic cerebellum, and vermis lesions often cause neuropsychiatric disorders. A study based on single photon emission computed tomography also suggested a functional impact of cerebellar lesions on cortical functioning through disruption of cerebellar-cerebral connections, indicating a role of the cerebellum in emotional processing [50]. Furthermore, abnormal cerebellar function was reported to be a potential marker of vulnerability to recurrent depression [51]. Based on these studies and our results showing abnormal white matter connections at the left middle frontal gyrus, the left limbic lobe uncus, and the right posterior lobe of the cerebellum, both the cortical-limbic circuit and the cerebellum may contribute to TRD.

Evidence from clinical findings supports the posterior lobe, rather than the anterior lobe, as the cerebellar region of specialization for cognitive and affective processes [52]. With cerebellar damage, there is a tendency toward lateralization in cognitive processing, and right-sided cerebellar lesions often show typical left-hemispheric dysfunctions, including disorders in executive functions, logical reasoning, and language skills [50]. Our results show white matter abnormalities in the left middle frontal gyrus, limbic lobe uncus, and right cerebellum posterior lobe are consistent with the tendency for lateralization [48]. The lateralized cerebral specialization is different between emotional experience and expression, and evidence suggests that positive, approach-related emotions are associated with functions of the left cerebral hemisphere regions, whereas negative, withdrawal-related emotions are associated with right hemisphere mechanisms [48]. Our results were focused on the left cerebral and right cerebellum posterior lobe, and suggest that TRD may be related to emotional-expression and cognitive-processing disorders.

Numerous studies have demonstrated that the cerebellum is involved in cognitive functions, especially the posterior lobe of the cerebellum, which is considered to be related to executive function, working memory, and language processing [46,47,50]. The left middle frontal gyrus is considered an important region for working memory, executive functions, logical reasoning, language skills, and information processing [50,53-55]. In our study, white matter abnormalities were observed simultaneously at the right posterior lobe of the cerebellum and the left middle frontal gyrus, which may enhance the impaired cognitive functions in TRD, and the poorer cognitive functions may be the basis of treatment resistance.

In one VBA study of eight refractory depression patients and nine controls, a significant reduction in FA was observed in the frontal lobe, ACC, and temporal lobe in depression patients [22]. However, the sample size of this study was small, and the results did not survive correction, with a threshold set by *P* < 0.005 and cluster size >30 voxels. In the present study using the same methods, a larger sample size, and a threshold of *P* < 0.001 and cluster size >50, we found that the cerebellum was also involved in the pathogenesis of TRD. Using the voxel-based method may produce Type I errors, although our results survived a FDR threshold of *P* < 0.05 at the cluster level or the voxel level.

We also found a negative correlation between depressive symptom scores and FA values in three areas in TRD patients, further supporting the possibility that damaged white matter integrity was related to disease severity. We did not find a correlation between FA values and age or disease duration, as previously reported [25,27]. These data suggest that reductions of FA in TRD may be related to patient clinical presentation, and less associated with other factors. There is also evidence that gender may influence white matter FA values [56]. However, we found no differences in FA between males to females in TRD patients in the three significant clusters, suggesting that male and female TRD patients exhibit the same pathogenesis.

There are some potential limitations of this study. First, the sample size is small, and these results require replication and further clarification in a larger patient population. Second, new methods such as TBSS should be used in the future to track the precise connections between the cortex, limbic area, and cerebellum, and to examine in more detail the network associated with affection regulation. The VBA as an explorative method is useful for discovering unanticipated or unpredicted neuroanatomical areas, although it can lead to pseudopositive results. Finally, more advanced statistical methods and a more powerful correction should be performed, as our small sample size limited the correction.

## Conclusions

Our DTI results demonstrate, for the first time, a role of the cerebellum in the pathogenesis of TRD. We suggest that the changes in white matter FA in TRD patients are similar to MDD, implicating abnormalities of the cortical-limbic circuits, but are also associated with the cerebellum. The pathogenesis of TRD may be related to abnormalities of cortical-limbic-cerebellar white matter networks. Future studies with larger sample sizes and better methods such as TBSS are required to replicate these results.

## Competing interests

The authors declare that they have no competing interests.

## Authors’ contributions

Authors HP and HZ designed the study and developed the protocols. LL and YN are tutors of HP. Authors HP, HZ, YZ, LZ, and HY carried out literature searches and analyses. Authors BS, JL, JZ, ZL, and ZZ performed statistical analyses and prepared the first draft of the manuscript. All authors read and approved the final manuscript.

## Pre-publication history

The pre-publication history for this paper can be accessed here:

http://www.biomedcentral.com/1471-244X/13/72/prepub
